# Supplementary material for: Opportunistic Non-Governmental Organisation Delivery of a Virtual Stop Smoking Service in England during the COVID-19 Lockdown
Source: Int J Environ Res Public Health. 2022 Jun 23;19(13):7722. doi: 10.3390/ijerph19137722 (PMC9266272; doi:10.3390/ijerph19137722)
Supplement: Supplementary file 1 [file ijerph-19-07722-s001.zip › S1 telephone interview schedule.pdf]

Supplementary S1: Telephone interview schedule

1: So far, how has this service matched your expectations in terms of providing support to help you quit smoking? PROMPT: what support did you think you would get and has this been provided?

2: What are your general impressions of this service? PROMPT: Ask for more detail on what they say if needed.

3. How does it compare to other services you've used in the past? PROMPT: If not used any other services just move on. PROMPT: Ask about whether had CO monitoring in the past and if so do they miss it.

4. Was there anything in particular about the service offered by Yorkshire Cancer Research that made you sign up to this rather than other stop smoking services that are available? PROMPT: Ask for more detail if give very short/closed response.

5. If you hadn't seen information about this service, do you think you would have sought out help elsewhere? If yes, where do you think you might have looked for support on quitting smoking. PROMPT: Ask ‘anywhere else’ to see if they give more info

6. Has the support provided helped you to quit or cut back on smoking? PROMPT: If not quit/cut back ask has the support helped in any other ways?

7. Are there any recommendations you can make for improving the service?

8. Do you have any other comments to make?
