# Supplementary material for: Opportunistic Non-Governmental Organisation Delivery of a Virtual Stop Smoking Service in England during the COVID-19 Lockdown
Source: Int J Environ Res Public Health. 2022 Jun 23;19(13):7722. doi: 10.3390/ijerph19137722 (PMC9266272; doi:10.3390/ijerph19137722)
Supplement: Supplementary file 1 [file ijerph-19-07722-s001.zip › S2 service cost estimates.pdf]

Supplementary S2: Service cost estimates

The costs provided in this section are intended to give a rough indication of the overall costs of the service. They are not based on length of time each participant engaged with the service or the specific stop smoking aids chosen by participants on an individual basis.

Promotion

The total cost of Facebook advertising was £356. This breaks down as a cost of:

- £3.80 per person that registered their interest in the service (n=94)
- £4.52 per person that engaged with the service and accepted support (n=79)
- £6.26 per person that achieved a 4-week quit (n=57)
- £7.00 per person that achieved a 12-week quit (n=51)

Postage

Postage costs totalled £1,218.00 across the 12 weeks of service delivery. Posting direct to participants ensured they had access to the stop smoking aids they had chosen to use such as NRT or e-cigarettes (including coils and liquids etc.)

SCP costs

The SCP costs are estimated from the costs obtained from details of the YESS Study (therefore will contain on-costs etc.). It is estimated that a SCP costs around £19.00 per participant per week. However, this is likely to be a slight overestimate given that the amount of time required per participant will drop over the weeks the service is delivered, with more intense support at the start with contact time reducing in length as the weeks progress.

NRT/e-cigarettes

The costs for stop smoking aids is estimated from the costs obtained from details of the YESS Study. For 12 weeks of supply the per person costs are estimated to be £142.44, the costs are broken down as follows (assumes use of both NRT and e-cigarette and therefore will be an overestimation as only some participants opted for both options):

- **Nicotine patches:**

- 21mg patches (6 weeks), £6.41 per box = £38.46
- 14mg patches (4 weeks), £9.88 per box = £39.52
- 7mg patches (2 weeks), £8.46 per box = £16.96
- **Total = £93.94**

- **E-cigarettes:**
  - E-cigarette (Innokin T18) = £15.00 (retail value £30.00)
  - Plug = £3.50
  - 12 coils (£1.50 each) = £18.00
  - 12 bottles of liquid (£1.00 each) = £12.00
  - **Total = £48.50**

Estimate service costs are summarised in the supplementary table below.

Supplementary table 1: Summary of service cost estimates

**Description**	**Per person per week**	**Per person total for 4 weeks**	**Total cost for 4 weeks (n=79)**	**Total cost per 4-week quit (n=57)**	**Per person total for 12 weeks**	**Total cost for 12 weeks (n=79)**	**Total cost per 12-week quit (n=51)**
Promotion	£4.52[a]	£4.52[a]	£356.89	£6.26	£4.52[11]	£356.89	£7.00
Postage[b]	£1.28	£5.14	£406.00	£7.12	£15.42	£1,218.00	£23.88
SCP[b]	£19.00	£76.00	£6,004.00	£105.33	£228.00	£18,012.00	£353.18
NRT/E-cigarette[b]	£11.87	£47.48	£3,750.92	£65.81	£142.44	£11,252.76	£220.64
**Total[c]**	**£36.67**	**£133.14**	**£10,517.81**	**£184.52**	**£390.38**	**£30,839.65**	**£604.70**

[a]The promotion costs are presented as a per person cost of recruitment and thus remain constant at 1, 4 and 12 weeks

[b]Costs are likely to be an overestimate as assumption made that each of 79 participants continue with quit aid and SCP support for full 12 weeks.

[c]Service costs for administration and estates have not been calculated.
